# Diagnostic accuracy of left ventricular longitudinal function by speckle tracking echocardiography to predict significant coronary artery stenosis. A systematic review

**DOI:** 10.1186/s12880-015-0067-y

**Published:** 2015-07-25

**Authors:** Ingvild Billehaug Norum, Vidar Ruddox, Thor Edvardsen, Jan Erik Otterstad

**Affiliations:** Department of Cardiology, Vestfold Hospital Trust, Pb 2168, 3103 Tønsberg, Norway; University of Oslo, Faculty of Medicine, Pb 1078 , Blindern, 0316 Oslo, Norway; Department of Cardiology, Oslo University Hospital, Rikshospitalet, Pb 4950, Nydalen, 0424 Oslo, Norway

**Keywords:** Coronary artery disease, Speckle tracking, Strain imaging, Left ventricular function, Chest pain

## Abstract

**Background:**

Patients evaluated for acute and chronic chest pain comprise a large, heterogeneous group that often provides diagnostic challenges. Although speckle tracking echocardiography (STE) has proved to have diagnostic value in acute coronary syndrome it is not commonly incorporated in everyday practice.

The purpose of the present systematic review was to assess the diagnostic accuracy of left ventricular (LV) longitudinal function by STE to predict significant coronary artery stenosis (CAD+) or not (CAD-) verified by coronary angiography in patients with chest pain suspected to be of cardiac ischemic origin.

**Methods:**

4 electronic databases; Embase, Medline, Cochrane and PubMed ahead-of print were searched for per 19.05.14. Only full-sized articles including > 40 patients were selected.

**Results:**

A total of 166 citations were identified, 16 full-size articles were assessed of which 6 were found eligible for this review. Of 781 patients included 397 (60 %) had CAD+. The overall weighted mean global longitudinal strain (GLS) was −17.2 % (SD = 2.6) among CAD+ vs. -19.2 % (SD = 2.8) in CAD- patients. Mean area under curve in 4 studies for predicting CAD+ ranged from 0.68 to 0.80. The study cut-off levels for prediction of CAD+ in the ROC analysis varied between −17.4 % and −19.7 % with sensitivity from 51 % to 81 % and specificity between 58 % and 81 %. In 1 study GLS obtained during dobutamine stress echocardiography (DSE) had the best accuracy. Regional strain measurements were not uniform, but may have potential in detecting CAD.

**Conclusions:**

GLS measurements at rest only have modest diagnostic accuracy in predicting CAD+ among patients presenting with acute or chronic chest pain. The results from regional strain, layer specific strain and DSE need to be verified in larger studies.

**Electronic supplementary material:**

The online version of this article (doi:10.1186/s12880-015-0067-y) contains supplementary material, which is available to authorized users.

## Background

In recent years it has become increasingly apparent that a large number of patients classified as low risk for acute coronary syndrome (ACS) and without diagnostic cardiac biomarkers represent the most prevalent group of patients admitted to hospital with chest pain [1–3]. In addition, we see numbers in our outpatient clinic referred for evaluation of stable chest pain. Up to 1/3 of patients with chest pain who are referred to coronary angiography have no significant coronary artery stenosis [4]. Though this investigation is generally safe, it has well known risk of complications and is also costly. Exercise testing is widely used for selecting patients for coronary angiography, but has its clear limitations as emphasized in the European guidelines for stable coronary artery disease [5]. In stable coronary artery disease, coronary computed tomography angiography (CTA) is a non-invasive alternative to assess coronary anatomy, but according to expert consensus only selected patients should be considered for CTA [5]. In guidelines concerning ACS [6] CTA is said to be useful to exclude ACS or other causes of chest pain, but due to limited availability and also the concern of radiation it is to our knowledge not commonly used in this setting worldwide. Thus we are in need of a simple, non-invasive method to improve the selection of patients who are referred to coronary angiography.

For several years measurements of left ventricular deformation, hereunder strain by speckle tracking echocardiography (STE), has been studied to evaluate global and regional left ventricular systolic function. Strain imaging has proven useful in several clinical settings i.e. evaluation of cardiotoxicity, ventricular function in heart transplant recipients and in acute coronary syndrome [7–11]. As pointed out by Feigenbaum [12], in spite of its feasibility and potential clinical value, yet only a few academic centers have incorporated it into everyday practice of echocardiography, and even then on a limited basis. Arguments being that STE is time consuming and provides results that are difficult to interpret. He advocates a simplified method incorporating solely measurements of longitudinal strain (LS) that might overcome some of these issues and make it easier to include STE in common clinical evaluation.

The purpose of this review is to evaluate the diagnostic accuracy of global (G) and regional (R) longitudinal strain to predict the presence or absence of significant CAD. A prerequisite for including studies was that all patients had undergone LS measurements and a subsequent coronary angiogram preferably < 2 months apart, subdividing them into significant coronary artery disease (CAD+) and no CAD (CAD-). The main objective was to assess the diagnostic accuracy of LS to predict CAD+ in these studies.

## Methods

We searched for studies that included patients who had been evaluated for significant CAD being related to chest pain, either in the acute setting such as suspected ACS, or electively with a question of stable angina pectoris. Previously known CAD may influence the strain-measurements and thereby represent a bias. Therefore, studies defining exclusion criteria for established CAD on presentation, such as a history of myocardial infarction (MI), previous percutaneous coronary intervention (PCI), open heart surgery or severe wall motion abnormalities were preferably included. We made some compromises leaving the possibility to include studies where a minority of patients had obvious CAD. Included studies should in principal also have excluded patients with overt heart failure, LV systolic dysfunction, atrial fibrillation or frequent ventricular premature complexes, also due to the possibility of influencing strain measurements.

In case of discrepancies in the assessment of inclusion or exclusion criteria, the problem was solved through consensus. We searched the following 4 electronic databases: Embase (1980 to 2014 week 20), Ovid MEDLINE (1946 to May 19.2014), Cochrane library and PubMed ahead-of-print as per May 19. 2014. The search strategy combined text words and subject headings identifying reports related to speckle tracking and angiography. The search strategy is presented in Additional file 1. In case of lacking data or questions of the methodology applied, the first authors were contacted for additional information. Only full text articles in the English language were accepted. Abstracts, reviews, letters to the editor, current opinions etc. were excluded from this review.

Consecutive series of > 40 patients who had been evaluated for significant CAD by left ventricular GLS and, eventually regional LS (RLS) measurements with a subsequent coronary angiography were included. CAD+ should have been defined as a minimum of at least one stenosis > 50 % or ≥70 % reduction of the arterial luminal area. Studies of LS at rest and, eventually during a stress test could be included. There should be a clear definition of how GLS and RLS were assessed. Provided LS measurements had been performed, studies with additional circumferential strain measurements were also included.

Selected studies should preferably present receiver operating characteristic (ROC) analysis for diagnostic accuracy including area under curve (AUC) values with an optimal cutoff and sensitivity and specificity of the strain method applied to predict CAD+. In principle, the LS values were compared between those with CAD+ vs. CAD-. The mean differences of the respective mean LS values in patients with CAD + vs. CAD- were calculated by simple subtraction for each study. For summary measures a plot depicting the mean values and SDs for GLS in both the CAD+ and the CAD- group from each study was constructed. A similar plot was constructed for the 4 studies that provided RLS data. We used STATA (version 12.0) and combined data from the different studies to make two virtual combined samples, CAD+ (*n* = 397) and CAD- (*n* = 381) respectively. We did not have access to raw data, but the mean and variance of the combined samples could be calculated from the reported values in the original studies. The mean GLS of the combined CAD+ sample, for instance, was calculated by taking the weighted mean of the reported GLS means of CAD+ patients from each study, the weights being each study’s relative size in the combined sample. The variances, and corresponding standard deviations, were found using standard formulas for pooled variance. A crucial implicit assumption in this process is that all studies we included used random samples from the same two populations (chest pain or other anginal equivalent and CAD- or CAD+) with no selection bias nor measurement bias being present in any of the studies (see “Discussion” for an elaboration on this point). We then assumed that LS values for CAD- and CAD+ are both normally distributed.

## Results

**Fig. 1:**
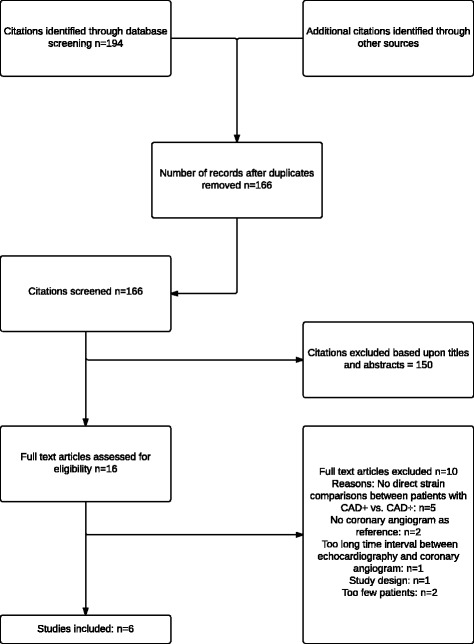
Flow diagram from a systematic search of 4 databases (MEDLINE,EMBASE, PubMed and Cochrane). Abbreviations: CAD+: Coronary artery disease. CAD-: No significant coronary artery disease. CAG; coronary angiogram

After database screening a total of 166 unique titles and abstracts were identified. A flow diagram of the studies selected is shown in Fig. 1 including principal reasons for excluding 10 full text articles [13–22]. A more detailed description explaining the arguments for exclusion of these studies (published between 2007 and 2014) is provided in Additional file 2. The six studies included [23–28] on basis of our predefined criteria were published between 2011 and 2014. No additional studies outside the search could be retrieved from screening reference lists in various reviews and full text articles.

### Description of individual studies

#### One study based upon the use of an Artida (Toshiba) system

**Table 1:** Layer-specific left ventrcular strain measurements at rest using CAG as reference for CAD

			Strain values (%)			Differences endo-epic. strain			
Study	Indication	CAG	CAD+	CAD-	*p*-value	CAD+	CAD-	*p*-value	AUC for CAD+, TLS/GCS (%)
Sarvari [23]	Suspected NSTE-ACS *n* = 77	CAD+ n = 49	TLS: −14.0 ± 3.3	−19.2 ± 2.2	<0.001	Δ2.4 ± 3.6	Δ5.3 ± 2.1	<0.001	Endocardial: 0.91/0.85
		CAD- n = 28	GCS: −19.3 ± 4.0	- 24.3 ± 3.4	<0.001	Δ2.4 ± 3.7	Δ10.4 ± 3.0	<0.001	Mid-myocardial: 0.91/0.87
			GLS: −15.3 ± 2.2	−19.2 ± 2.2	<0.001	Δ2.4 ± 3.8	Δ5.3 ± 2.1	<0.001	Epicardial: 0.79/0.68

Strain values and differences between andocardial and epicardial strain are presented as mean ± SD

*NSTE-ACS* Non-ST elevation acute coronary syndrome, *CAG* Coronary angiography, *CAD+* Coronary artery disease, defined as coronary artery stenosis ≥ 50 %, *TLS* Territorial longitudinal strain, *GCS* Global circumferential strain, *endo* endocardial, *epic* epicardial, *AUC* Area under the curve for ROC analysis of prediction of CAD being present or not

In the study of Sarvari et al. [23] 77 patients were prospectively referred to a single tertiary coronary care center with suspected non-ST-elevation acute coronary syndrome (NSTE-ACS) for coronary angiogram. Exclusion criteria were preexisting CAD, defined by a history of previous MI, PCI and open chest surgery. Echocardiography was performed 1–2 h prior to the invasive procedure and within 48 h after the last episode of chest pain. LV strain was measured from a parasternal short-axis view of the LV at the level of the papillary muscle for circumferential strain and 3 apical views for LS. The Artida system allows analysis of the 2D images for endocardial, mid-myocardial and epicardial strains.

Peak negative systolic strains from the 3 layers in 16 longitudinal LV segments were averaged from each myocardial layer to calculate GLS. Layer-specific strain measurements were also used for RLS. RLS was calculated based on the perfusion territories of the 3 major coronary arteries in a 16 segment model, modified according to Cerquieira et al. [29], by averaging all segmental peak systolic strain values within each territory and termed territorial longitudinal strain (TLS) in the article.

Global circumferential strain (GCS) was measured for each myocardial layer from one parasternal short axis view (papillary muscle level) in 6 circumferential LV segments. Wall motion was visually assessed and a 16 segment model and wall motion score index was calculated for each patient as the average of the segmental values. Data for endocardial strain values only is presented in Table 1.

Patients with CAD+ had worse function in all 3 myocardial layers assessed by GLS and GCS compared with CAD- patients. The average differences between CAD+ and CAD- patients from the endocardial layer were 3.9 % for GLS; 5.2 % for RLS, and 5.0 % for GCS. The absolute differences between endocardial and epicardial strain values were significantly lower in patients with CAD+ than in those with CAD- (Table 1). ROC analysis revealed high AUC values for endocardial and mid-myocardial RLS and GCS compared to those obtained from the epicardial layers (*p* < 0.05), from wall motion score index (*p* < 0.01) and LVEF (*p* < 0.001). The optimal cut-off value for identifying patients with CAD + was −16.4 % for endocardial RLS with a sensitivity/specificity/positive (PPV) and/negative (NPV) predictive value of 89 %/81 %/73 %/93 % respectively. No such detailed data is presented for GLS in the publication. With multivariate regression analyses, including parameters influencing myocardial function, endocardial RLS (per1% change) was the only predictor of the presence of CAD+ (OR 2.10, 95 % CI 1.5 – 3.1, *p* < 0.001).

In conclusion, assessment of layer-specific strain by 2D STE might identify NSTE-ACS patents with CAD. Endocardial function was found to be more affected in CAD+ compared with epicardial function and LVEF, with endocardial and mid-myocardial RLS having the best diagnostic accuracy.

#### 4 studies based upon GE equipment at rest

##### Elective diagnostic coronary angiography

Montgomery et al. [24] retrospectively studied 2D STE characteristics in 123 consecutive patients who underwent stress echocardiography, and subsequently coronary angiography within 10 days. In this study 23 patients had been excluded due to obvious CAD, LVEF < 40 % or severe wall motion abnormalities. However a total of 9 subjects (7 %) included had prior revascularization procedures (five CABG).

The diagnostic power of GLS at rest was compared with the wall motion score index during stress for detecting CAD+. STE was performed on all 3 apical views at rest and mean GLS was calculated. For RLS peak LAD segmental strain measurements were compared, and the RLS values presented in Table 2 express LAD segmental peak systolic strain as well as the ROC analysis for GLS and RLS. The mean differences in GLS and RLS between CAD+ and CAD- patients were 2.3 % and 2.7 % respectively. In the ROC analysis the sensitivity/specificity of the strain cut-off points listed in Table 2 were comparable to a cut-off point for wall motion score index ≥ 1.13 (68 %/70 %) measured during stress. In conclusion GLS measurements at rest did not differ from wall motion score index during stress for identification of patients with CAD+. The study did not incorporate strain measurements during stress.

**Table 2:** 4 studies on measurements of left ventricular strain at rest to predict CAD+ using coronary angiogram as reference

Study	n	CAG	GLS (%)	p CAD+ vs CAD-	TLS (%)	pCAD+ vs CAD-	AUC for CAD+ (95 % CI)	Cut-off	Sensitivity	Specificity
Montgomery [24]	123	CAD+ n = 56^a^	−16.8 ± 3,2				GLS 0.72 (0.63-0.82)	- 17.8 %	66 %	76 %
		CAD- n = 67	−19.1 ± 3.4	0.0002			TLS 0.73 (0.62 - 0.83)	- 18.3 %	69 %	70 %
	109	LAD+ n = 35^c^	−19 ± 2.8		−18.68 ± 3.3					
		LAD- n = 74	−17.5 ± 2.7	0.0001	−21.14 ± 3.3					
Smedsrud [25]	86	CAD+ n = 43^a^	−17.7 ± 3.0		−17.9 ± 3.5		GLS 0.68 (0.56-0.79)	−17.4 %	51 %	81 %
		CAD- n = 43	−19.5- ± 2.6	0.003	−20.1 ± 2.9	0.015	TLS 0.67 (0.52-0.82)	n.a.	n.a.	n.a.
Biering-Sørensen [26]	293	CAD+ n = 107^b^	−17.1 ± 2.5^b^				GLS 0.68 (0.62-0.74)^d^	- 18.4 %	74 %	58 %
		CAD- n = 186	- 18.8 ± 2.6	<0.001						
Shimoni [27]	97	CAD+ n = 69^a^	−17.3 ± 2.4		−9.1 ± 3.2		GLS 0.80	−19.7 %	81 %	67 %
		CAD- n = 28	−20.8 ± 2.3	<0.001	−12.9 ± 2.3	<0.001	TLS 0.75	−12.6 %	77 %	68 %

Abbreviations and definitions: CAD+ was defined as ≥ 50 % stenosis ^a^ in one or more coronary arteries and as ≥ 70 % luminal area reduction^b^

LAD: Left anterior desending ^c^subset of 109 patients where TLS in the LAD territory was performed. ^d^ GLS from 12 segments. TLS = territorial longitudinal strain, defined specifically in the text from each study

Smedsrud et al. [25] included 86 patients and investigated whether the duration of LV early systolic lengthening could accurately identify patients with CAD+. Since LS measurements also were performed, the study was included in the present review. Consecutive patients were referred to elective coronary angiography because of stable chest pain. Echocardiography was performed immediately before coronary angiography. Exclusion criteria were ACS, a history of MI and/or any evidence of scar by late enhancement on contrast-enhanced MRI or previous heart surgery. GLS was measured from the 3 apical views model and was based upon the peak negative systolic strain value. RLS was calculated as the average of the segments belonging to each perfusion territory of the 3 major coronary arteries [29]. The average difference between patients with CAD+ and CAD- was 1.8 % and 2.2 % for GLS and RLS respectively, In the ROC analysis GLS showed poor sensitivity for predicting CAD+ (Table 2), with a PPV of 0.73 (95 % CI 0.57-0.86) and NPV of 0.63 (95 % CI 0.54-0.69). In contrast to the modest diagnostic accuracy of GLS and RLS the prolonged duration of systolic lengthening showed a far better accuracy with an AUC of 0.83 (95 % CI 0.75-0.92) for predicting CAD+.

In a large study Biering- Soerensen et al. [26] enrolled 296 consecutive patients with suspected stable angina pectoris. 3 patients were excluded due to poor quality of the echocardiographic images. The remaining 293 participants were all examined with echocardiography, including 2DSE and exercise test followed by coronary angiography (median 25 days with interquartile range 14–45 days (T. Beiering-Soerensen, personal communication). Exclusion criteria were known ischemic heart disease, congestive heart failure, heart valve disease, LVEF < 50 %, intraventricular conduction disturbances, pathological Q-waves and arrhythmias. This is the only study where CAD+ was defined as stenosis with ≥ 70 % reduction of the arterial lumen, which corresponds to 50 % reduction in arterial diameter. GLS was calculated from the average of 18 segments (GLS^18^) as well as from 12 segments (GLS^12^), derived from 6 basal and 6 midventricular segments, thereby excluding the 6 apical segments. RLS was termed regional peak systolic strain and measured in 18 segments from all views. Multiple regression models were constructed and significant stenosis in the LAD, RCA and LCX were tested as independent predictors of RLS in each of the 18 segments. The typical disitribution of these three arteries were adopted from Lang et al. [30], incorporating that some segments have variable coronary perfusion. RLS values are not reported directly in the article.

GLS^12^ data is presented in Table 2, since this was the only independent predictor of CAD+ remaining after multivariate adjustment. The mean difference in GLS^12^ between patients with CAD+ vs. CAD- was 1,7 %. As presented in Fig 1 in the Data Supplement of the article, RLS was significantly lower in segments supplied by stenotic coronary arteries compared with nonstenotic arteries in a pattern closely mimicking the anatomic perfusion area. When the data from GLS^12^ and the exercise test was combined in ROC analysis, the AUC was significantly higher than that for the exercise test alone (0.84 versus 0.78, *p* = 0,007.). In conclusion GLS^12^ peak systolic strain at rest was found to be an independent predictor of CAD+ and significantly improved the diagnostic performance of exercise testing.

##### Patients hospitalized with acute chest pain

The study of Shimoni et al. [27] included 97 consecutive patients, hospitalized with chest pain and suspected CAD, who had LVEF > 50 % and normal regional LV systolic function. Patients with ST-elevation MI at presentation or a history of myocardial infarction were excluded. This study was included by consensus among the authors, since 21 patients (22 %) had previous revascularization. A control group of 51 patients referred to stress echocardiography for evaluation of atypical chest pain was not evaluated in our review, because they had no coronary angiography. GLS was measured from the apical views. RLS was computed as that segment with the least negative strain value during systole using the segmental division of the three major coronary arteries introduced by Cerquira et al. [29]. Although this study incorporated a histogram with different variation in strain measurements, the data included in Table 2 is based upon peak systolic strain values obtained from 16-segment model. The mean difference between patients with CAD+ vs- CAD- was 3.5 % for GLS and 3.8 % for RLS. The ROC analyses revealed higher AUC values than the three other studies (Table 2).

#### One study incorporating strain measurements at rest and during dobutamine stress (GE equipment)

In the study of Ng et al. [28] 177 consecutive patients were identified from a database as had been evaluated for stable CAD. Exclusion criteria was LVEF < 40 % or moderate to severe valvular disease. The time lapse between echocardiography and coronary angiography was up to 6 months, and 30 % of the patients had a history of MI. However, in view of its interesting findings including strain measurements during dobutamine stress echocardiography (DSE) the study was included as a compromise. A derivation study population that comprised 62 patients and a validation study population of 40 patients underwent clinically indicated DSE performed < 8 days before coronary angiography. Patients with AMI < 4 weeks, heart failure, cardiomyopathy or severe valvular heart disease had been excluded. The mean difference for GLS in the derivation group was 2.8 % as opposed to 6.0 % during peak stress with the respective figures in the validation group being 1.5 % and 3.0 %. The diagnostic accuracy as judged from the ROC analysis of GLS at peak stress in the derivation group was excellent with an optimal cutoff at - 20.0 (Table 3), being similar to wall motion score index. No such data was presented from the validation group, and RLS was not measured in this study. In conclusion the introduction of GLS strain measurements during DSE resulted in a far better diagnostic accuracy for CAD than of the resting values, especially in the derivation subgroup.

**Table 3:** GLS measurements at rest and during PDS for identification of significant CAD on CAG

Study	Strain masurements	Derivation group, n = 62		p-value	Validation Group n = 40		*p*-value
		CAD- n = 14	CAD+ n = 48		CAD- n = 15	CAD+ n = 25	
Ng [28]							
	GLS at rest	−19.1 ± 2.9	−16.3 ± 2.4	0.001	−19.0 ± 2.8	−17.5 ± 2.4	n.s.
	GLS at PDS	−21.7 ± 3.0	−15.7 ± 2.9	<0.001	−20.7 ± 0.8	−17.7 ± 2.7	<0.05
	ROC analysis		Optimal cut-		Sensitivity	Specicity	Accuracy
		AUC	off for GLS		(%)	(%)	(%)
	Peak stress	0.93, *p* < 0,001	−20 %		84	88	85

mean ± SD

*n.s* not significant, *PDS* Peak dobutamine stress, otherwise as in Table 1

**Table 4:** Table showing the combined mean and pooled SD at rest in the CAD - and CAD + populations respectively as each study included is added

CAD +							CAD -					
Study	n (% of total weight)	Cumulative n	Mean GLS, %	SD	Combined mean	Pooled SD	n (% of total weight)	Cumulative n	Mean GLS, %	SD	Combined Mean	Pooled SD
Ng	48 (12 %)		−16,3	2,4			14 (4 %)		−19,1	2,9		
Ng	25 (6 %)	73	−17,5	2,4	−16,7	2,5	15 (4 %)	29	−19	2,8	−19,0	2,8
Montgo	56 (14 %)	129	−16,8	3,2	−16,7	2,8	67 (18 %)	96	−19,1	3,4	−19,1	3,2
Smedsr	43 (11 %)	172	−17,7	3	−17,0	2,9	43 (11 %)	139	−19,5	2,6	−19,2	3,1
Shimon	69 (17 %)	241	−17,3	2,4	−17,1	2,8	28 (7 %)	167	−20,8	2,3	−19,5	3,0
B-Sørens	107 (27 %)	348	−17,3	2,5	−17,1	2,7	186 (49 %)	353	−18,9	2,6	−19,2	2,8
Sarvari	49 (12 %)	397	−17,3	2,2	−17,2	2,6	28 (7 %)	381	−19,2	2,2	−19,2	2,8

The study by Ng has two lines because it was divided into one validation and one derivation group

Abbreviations: *CAD+* coronary artery disease, *CAD-* no coronary artery disease, *SD* standard deviation, *GLS* slobal longitudinal strain

Cumulative n describes the total number of pateints as each study is added on

## Summary of results

**Fig. 2:**
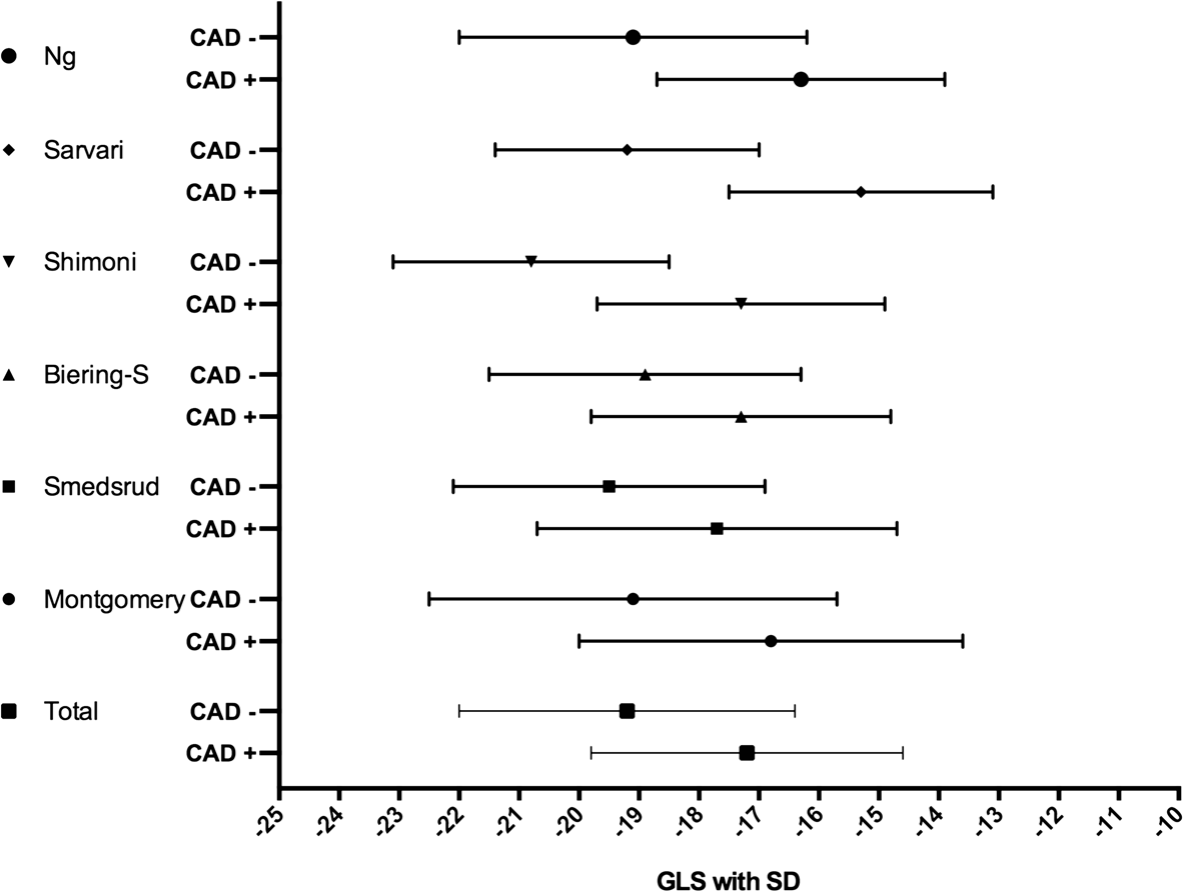
Six included studies with results of mean GLS with SD in their CAD +/CAD- groups. Abbreviations: CAD+: Coronary artery disease. CAD-: No significant coronary artery disease. GLS: Global longitudinal strain. SD: standard deviation

In these 6 studies 778 patients with suspected CAD were examined with speckle tracking echocardiography and coronary angiography; 397 (51 %) had CAD+ and 381 (49 %) had CAD- judged from the angiographic criteria applied. The majority, of patients included had no evidence of obvious CAD on presentation. The most uniform measurements were GLS at rest. Table  4 shows the combined mean and pooled SD in the CAD+ and CAD- populations respectively as each study included is added.The diagram in Fig. 2 shows the differences in GLS measured at rest in the CAD+ and CAD- patients in the six studies and the combined mean and SD for the six studies together. GLS values from the study of Sarvari et al. [23] have been assessed from the endocardial layer. Summarizing these results by methods previously described, patients with CAD+ (*n* = 397) had on average GLS −17.2 (±2.6)% as opposed to −19.2 (±2.8)% among those with CAD- (*n* = 381). The average mean difference in GLS between the two groups was 2.7 (interstudy range 1.7-3.9).

**Fig. 3:**
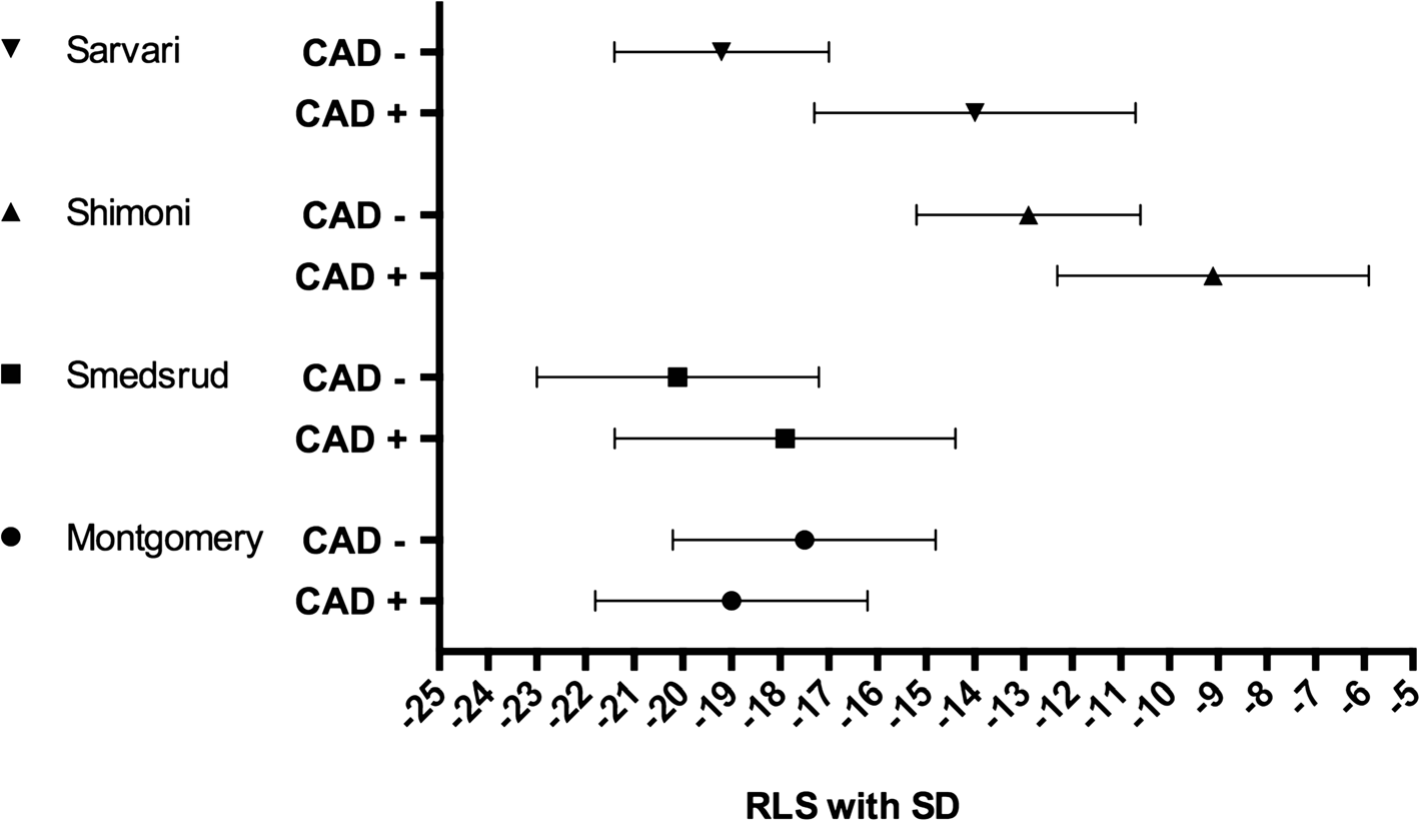
Four included studies with results of mean RLS with SD in their CAD +/CAD- groups. Abbreviations: CAD+: Coronary artery disease. CAD-: No significant coronary artery disease. RLS: regional longitudinal strain. SD: standard deviation

Although 4 studies reported RLS measurements, the heterogeneous methodology applied did not allow any direct inter-study comparison. The individual studies and their respective mean and SD for RLS in the CAD+/CAD- groups are presented in Fig. 3. In two of the studies ROC analysis of GLS at rest was not presented [23, 28].

The area under curve in 4 studies for predicting CAD+ ranged from 0.68 to 0.80. The cut-off level for prediction of CAD+ in the ROC analysis varied between −17.4 % and −19.7 % with a sensitivity from 51 % to 81 %) and specifcity between 58 % and −81 %.

## Discussion

In this review of a limited number of patients recruited from 6 heterogeneous studies, measurements of GLS and RLS revealed significantly higher values (less negative) among patients with CAD+ than in those with CAD-. However, the overlap in distribution of GLS values between the CAD+ and CAD- groups was considerable both in the individual studies and in our analysis of the combined samples. This implies that it is difficult to correctly identify a patient as CAD+ or CAD- using GLS values at rest alone, and stresses the point that statistical significance does not equal clinical relevance.

It should be noted that our analysis of the combined samples does present some methodological problems. It assumes implicitly that all studies used the same inclusion criteria, and that no selection bias was present in any of the studies. This is a problem with many combined analysis of several studies and also in this case. Furthermore, it disregards various possible measurement biases that may stem from the use of different equipment and measurement methods and/or skills. Also, the assumption of normality in distribution of GLS values for CAD+ and CAD- patients may or may not hold (without access to raw data, this assumption could not be subjected to statistical tests).

We still argue that even with these challenges present, the analysis still provides useful insight as to the clinical relevance of GLS at rest in this context. This is because the estimated degree of overlap between GLS values of CAD+ and CAD- patients is so significant that the overall picture would most likely remain the same even if several possible biases were adjusted for (note also that the individual studies produce similar degrees of overlap). As for the assumption of normality, the significant degree of overlap does not rely on the distributions being exactly normal, as is evident in Fig. 2. Substantial changes to skewness and/or kurtosis will still produce overlap with the same clinical implications. Hence we argue that the value of GLS at rest to identify CAD+ patients with chest pain from those with CAD- is only moderate.

Interestingly, in one study [28] a subgroup that underwent DSE demonstrated that this overlap was nearly eliminated when GLS was calculated during peak stress. On the other hand, such a difference was not as pronounced in another subgroup that underwent DSE in the same study. In one study [23], using layer specific measurements of both GLS, RLS and circumferential strain at rest, a very high diagnostic accuracy was observed for the endocardial and mid-myocardial layers.

Since CAD is primarily a regional problem and GLS is a global assessment the present findings were perhaps not surprising. The study of Sarvari et al. [23] using layer-specific regional strain and the study of Shimoni et al. [27] using the least negative strain value during systole for the segmental division of three major coronary arteries introduced by Cerquira et al. [29] both showed a lesser degree of overlap of the strain values in the CAD+/CAD-groups and thereby better potential (Fig. 3). However, in the two other studies that reported RLS, the overlap was significant and did not appear to be better than for GLS.

The different methods used for obtaining regional strain results represent a problem. In 1989 the American Society of Echocardiography recommended a 16-segment model for LV segmentation [31]. In 2002 they introduced a 17-segment model in an attempt to establish segmentation standards applicable to all types of imaging. The new segment was the apical cap [29]. This model should be used for myocardial perfusion studies and the 16-segment model is appropriate for studies assessing wall-motion abnormalities. The Bulls-Eye used for RLS assessment is derived from the latter for both GE and Toshiba equipment. In the 2002 recommendations individual segments were clustered and combined assigned to specific coronary artery territories, although there is individual variability in the coronary blood supply to myocardial segments. Most studies in this review including RLS have incorporated this model, but two studies used the average of all segments in one territory [23, 25], one used the lowest strain value a single segment [27], one assessed the average values in the LAD segment only [24] and one did not incorporate RLS measurements at all. In the large Danish study [26], in contrast to the others, ischemic segments were not assigned to prespecified vascular territories because they obtained information about how significant stenosis in the three main coronary arteries affected regional LS in each segment.

Until a standard and well established approach is generally adopted, measurements today of RLS is hampered by the use of an arbitrary anatomical model that does not necessarily reflect the individual coronary artery distribution, and the need for proper standardization of how to uniformly obtain RLS data is obvious.

Patients studied were principally without LV dysfunction, overt heart failure, left bundle branch block or heart failure, and only a minority had evidence of previous CAD, most of whom had been revascularized. With that reservation in mind, the results observed are probably representative for most patients presenting with chest pain and without previously known CAD.

It seems to be that the number of patients hospitalized for non-coronary chest pain (NCCP) is increasing [1–3]. This group consists of patients both with and without established CAD. Interestingly, only two of the studies [23, 27] evaluated LS measurements in patients hospitalized for acute chest pain. The 56 CAD- patients in these studies most probably represent NCCP. Dahlslett et al. recently published a study that explored the value of LS measurements at rest and during stress to predict the absence of CAD [22]. Because the study was designed to “rule out” CAD rather than detect it, it was not included in this review. Interestingly they found that strain measurements may be helpful in excluding significant CAD among NCCP patients. Clearly, larger “rule out studies” are needed in order to further evaluate the role of LV strain measurements this increasing group of patients that represent a huge burden to contemporary health care. There are at present no established guidelines for management of such patients, and a simple, fast method to exclude those without CAD beyond the information obtained from ECG and cardiac markers would be highly welcome. Strain measurements by STE at rest are fast and simple to apply, without any discomfort to the patients. If this method appears to be of value in the classification of patients with unexplained chest pain to CAD+ or CAD- it may obtain acceptance for a more common use in clinical practice.

The results from this review, however, were somewhat disappointing both in the acute and elective evaluation of suspected CAD, confirming that GLS at rest is not sensitive enough to detect regional CAD. The influence of afterload and diastolic function on GLS may have contributed to these findings, but these parameters were not addressed in this review.

The overlap may be overcome by the introduction of layer specific measurements [23].

For patients with stable CAD the addition of STE to simple stress test that is fast and easy to perform may be of value in this context. The improved diagnostic accuracy of an ordinary exercise test added to GLS measurements in the study of Biering-Sørensen et al. [26] is interesting, but requires considerable time and resources. The findings of Ng et al. [28] were somewhat disturbing, with different diagnostic accuracy of DSE with strain in the two subgroups studied. The use of DSE with strain is further hampered by the limited availability of DSE in many smaller hospitals. This procedure requires highly trained staff, good machine capacity and a considerable volume of examinations. Therefore, a proven clinical value of strain measurements at rest would be highly beneficial to many cardiologists in everyday practice. As with exercise testing, adding pretest probability is essential and a model including this and loading conditions along with strain measurements would possibly improve the benefit of STE in clinical practice.

### Study limitations

Selection bias is present in studies selected for this review, represented by the different number of patients with obvious CAD on inclusion. Knowledge of previous CAD might influence the measurements because the technique is not fully automated and thereby operator dependent. The presence of established CAD would require previous strain images for comparison, like the assessment of a new ECG requires old ECGs for the evaluation of new changes in patients with established CAD. At the present stage, it seems unrealistic to assume that most patients with CAD have previous strain measurements for comparison. Therefore, we aimed to comprise patients without previously established CAD when admitted with chest pain.

It ought to be emphasized that we only included studies where coronary angiography was performed, hence selection bias is presented and the results cannot be applied to the large group of patients with chest pain and CAD ranging from stable angina to ACS.

In addition, selection bias may be unavoidable when different selection criteria for “good quality images” are being applied. Due to operational bias, better results may be expected in studies with the most experienced and competent echocardiographers. Differences in the time lapse between the two examinations may also have an influence across studies, not only within them.

Most studies had used GE Vingmed equipment. The problem of manufacturer bias is already introduced with the inclusion of the study using Artida Toshiba system, and more will follow. It will present a challenge for the medical community and the various manufacturers to introduce uniform principles for strain measurements in the future. Operator bias in the review cannot be excluded, since no study reported the level of expertise of the actual Using too strict inclusion criteria for studies may have precluded this present review. Therefore, a more extended validation of the strain method may have been missed. Only 15 full articles were assessed after the performing the previously described search and there is always a chance that important studies were left out of although no additional citations could be found from screening the reference lists of these articles and reviews included in the search.

## Conclusions

Based upon the findings GLS measurements at rest only have modest diagnostic accuracy in predicting CAD+ among patients presenting with acute or stable chest pain. GLS measurements during peak DSE and showed better accuracy in two separate studies, but these findings have to be verified in larger studies. RLS showed greater potential in detecting CAD, but the heterogeneity for obtaining regional data and different terminology remains a challenge. Clearly, more refined strain measurements at rest are needed to identify patients with vs. without significant coronary heart disease, and the introduction of models including pretest probability and possibly also afterload/diastolic function should be developed.

## Additional files

Additional file 1:
**Search strategy.**
Additional file 2:
**Reasons for excluding 10 full text articles.**

## Abbreviations

ACS: Acute coronary syndrome
AUC: Area under curve
CAD+: Significant coronary artery stenosis
CAD-: No significant coronary artery stenosis
CTA: Coronary computed tomography angiography
DSE: Dobutamine stress echocardiography
GCS: Global circumferential strain
GLS: Global longitudinal strain
LS: Longitudinal strain
LV: Left ventricular
MI: Myocardial infarction
NCCP: Non coronary chest pain
NPV: Negative predictive value
PCI: Percutaneous coronary intervention
PPV: Positive predictive value
ROC: Receiver operating characteristics
SD: Standard deviation
STE: Speckle tracking echocardiography
RLS: Regional longitudinal strain
