# Supplementary material for: Diagnostic accuracy of left ventricular longitudinal function by speckle tracking echocardiography to predict significant coronary artery stenosis. A systematic review
Source: BMC Med Imaging. 2015 Jul 25;15:25. doi: 10.1186/s12880-015-0067-y (PMC4513709; doi:10.1186/s12880-015-0067-y)
Supplement: Additional file 1: — Search strategy. [file 12880_2015_67_MOESM1_ESM.doc]

Database(s): **Embase** 1980 to 2014 Week 20
Search Strategy:

| **#** | **Searches** | **Results** |
| --- | --- | --- |
| 1 | angiocardiography/ | 73511 |
| 2 | ((coronar* adj3 (angiograph* or angio-graph*)) or angiocardiograph* or angio-cardiograph*).tw. | 43278 |
| 3 | or/1-2 | 85705 |
| 4 | (speckl* adj3 track*).tw. | 4946 |
| 5 | 3 and 4 | 160 |

Database(s): **Ovid MEDLINE(R) In-Process & Other Non-Indexed Citations and Ovid MEDLINE(R)** 1946 to Present
Search Strategy:

| **#** | **Searches** | **Results** |
| --- | --- | --- |
| 1 | Coronary Angiography/ | 48371 |
| 2 | ((coronary adj3 (angiograph* or angio-graph*)) or angiocardiograph* or angio-cardiograph*).tw. | 32390 |
| 3 | 1 or 2 | 64214 |
| 4 | (speckl* adj3 track*).tw. | 1684 |
| 5 | 3 and 4 | 41 |

Cochrane Library - Last Saved: 21/06/2013 08:37

| ID | Search |  |
| --- | --- | --- |
| #1 | speckl* near/3 track*:ti,ab,kw | 77 |
| #2 | ((coronary near/3 (angiograph* or angio-graph*)) or angiocardiograph* or angio-cardiograph*):ti,ab,kw | 5119 |
| #3 | #1 and #2 | 2 |

PubMed

| # | Search |  |
| --- | --- | --- |
| 1 | (((speckl* AND track*) AND ((((angiograph*) OR angiography) OR angiocardiograph*) OR angio-cardiograph*))) AND publisher[sb] | 1 |
