# Supplementary material for: Diagnostic accuracy of left ventricular longitudinal function by speckle tracking echocardiography to predict significant coronary artery stenosis. A systematic review
Source: BMC Med Imaging. 2015 Jul 25;15:25. doi: 10.1186/s12880-015-0067-y (PMC4513709; doi:10.1186/s12880-015-0067-y)
Supplement: Additional file 2: — Reasons for excluding 10 full text articles. [file 12880_2015_67_MOESM2_ESM.docx]

**Additional file 2 – reasons for excluding 10 full text articles**

*No strain comparison between patients with CAD+ vs. CAD- on CAG, n = 5*

- Grenne et al [13] consecutively assessed myocardial function in 102 patients with suspected non-ST elevation (NSTE) ACS awaiting CAG. Patients without evidence of significant CAD were classified as having noncoronary chest pain (NCCP). The authors reported impaired GLS and GCS in patients with NSTEMI compared with NCCP. They compared CAG findings among NSTEMI patients with occluded vs. non-occlusive CAD. The study did not incorporate any direct strain comparison between those with CAD+ vs.CAD÷ on CAG. In addition, the study included patients with a history of MI (14%) and previous PCI (15%)
- In another study by Grenne et al [14] 111 patients with suspected NSTE-ACS, and patients with a history of MI and open chest surgery were not included. Patients with NCCP were also studied with CAG, but this study compared 21 patients with NSTEMI and acute coronary occlusion with 90 patients with non-occluded arteries, representing clinical diagnoses of NSTEMI, UAP and NCCP.
- In a study by Eek et al [15] patients were prospectively enrolled and included with a clinical diagnosis of NSTE-ACS of whom 5% had a history of CAD. Patients with NCCP were not included and CAG findings were compared among NSTE-ACS patients with and without coronary

occlusions.

- In the study of Choi et al [16] consecutive patients were evaluated for belonging to a high-risk, low-risk and a control group without CAD. No comparison between high+low risk vs. controls was reported.
- Clear-cut comparions for GLS or TLS are difficult to detect in the comprehensive study by Celutkiene et al [17], in which 47% of patients had known or suspected CAD. CAG was performed 6-8 weeks after the dobutamine challenge.

*Studies without CAG as reference, n = 2*

- The study of Stanton et al ]18] on the mortality prediction of GLS did not incorporate GAC in 546 unselected consecutive individual undergoing clinically indicated echocardiography. A history of MI was reported in 35%, and previous PCI or CABG in 18% ofpatients included.
- Nucifora et al [19] included 182 patient referred to multisclice computed tomography (MSCT) coronary angiography and echocardiography including GLS measurements. Patients with a history of CAD, overt LV systolic dysfunction (LVEF < 50%) or LV wall motion abnormalities had been excluded. They compared 3 groups based upon the MSCT coronary angiography findings: No CAD, nonobstructive CAD and obstructive CAD. Accordingly, no direct CAD+ vs. CAD- comparisons were reported.

*Too long time span between echocardiography and subsequent CAG, n = 1*

- Hanekom et al [20] studied consecutive patients who underwent both DSE for clinical evaluation of myocardial ischemia and CAG within 12 months of DSE. Although patients with severely depressed LV function and previous CABG were excluded, a substantial number of patients may have had preexistent CAD, and 19% had Q-waves in their ECGs. Strain results are not clearly expressed as GLS or TLS.

*Too few patients*

- Anwar et al [21] enrolled 25 patients to coronary angiography and speckle tracking echocardiography. The majority obviously had preexisting coronary heart disease as reflected from 10 patients having angina pectoris NYHA class III, with multiple cardiovascular risk factors, 5 a positive stress ECG, 4 typical chest pain at rest with accompanying ECG changes, 4 atypical chest pain with impaired LV systolic function by 2D echo and 2 atpical chest pain following CABG.

*Study design:*

- Dahlslett et al [22] enrolled 64 patients with suspected NSTE-ACS. The objective was to investigate whether myocardial strain assessment could exclude significant coronary artery disease in case of abscnsce of ischaemia on ECG and negative biomarkers. Interestingly they found that myocardial strain measurments might be helpful in this respect. However, the study was designed to exclude CAD rather than to detect it, and thus was not included in this review.
